# Multitarget protection by Chinese herbal medicines against sensorineural hearing loss: a focus on oxidative stress, apoptosis, and inflammation

**DOI:** 10.3389/fmed.2026.1785210

**Published:** 2026-03-20

**Authors:** Yu Tian, Aiping Wang, Hui Leng

**Affiliations:** 1 The First Clinical College, Liaoning University of Traditional Chinese Medicine, Shenyang, Liaoning, China; 2 Department of Otolaryngology, Head and Neck Surgery, Affiliated Hospital of Liaoning University of Traditional Chinese Medicine, Shenyang, Liaoning, China

**Keywords:** hearing impairment, mechanism, sensorineural hearing loss, therapeutic effect, traditional Chinese medicine

## Abstract

**Objective:**

Based on the molecular mechanisms of sensorineural hearing loss (SNHL), this study aims to explore the role of traditional Chinese medicine (TCM) in the treatment of SNHL, promote the development of deafness-related research, provide new insights for the formulation and medication of TCM in the treatment of SNHL, and further facilitate the better development of new drugs for clinical application.

**Methods:**

Relevant literatures on sensorineural hearing loss (SNHL) and its treatment with traditional Chinese medicine (TCM) were reviewed. The pathogenesis of SNHL and the related research achievements of TCM in treating this disease were sorted out, and the existing research conclusions were analyzed and summarized.

**Results:**

Sensorineural hearing loss (SNHL) is a type of hearing impairment mainly caused by damage to cochlear hair cells, spiral neurons, and/or the auditory center, including drug-induced deafness, noise-induced deafness, and age-related deafness. Its molecular mechanisms mainly involve apoptosis, oxidative stress injury, immune inflammation, and metabolic disorders. Due to the irreversibility (non-regeneration) of hair cells and spiral neurons, there is currently no effective treatment for hearing loss caused by such damage. In recent years, the use of traditional Chinese medicine (TCM) to treat hearing disorders by regulating the above-mentioned molecular mechanisms has gradually become a research hotspot, and a large number of studies have confirmed that TCM exhibits significant efficacy in hearing treatment.

**Conclusion:**

Traditional Chinese medicine (TCM) can exert a therapeutic effect on hearing by regulating the core molecular mechanisms of SNHL (such as apoptosis and oxidative stress injury) and has significant application potential. The discussion in this article can provide a new direction for the formulation and medication of TCM in the treatment of SNHL, and has positive significance for promoting the development of deafness research and the research and development of new clinical drugs.

## 1 Introduction

Sensorineural hearing loss (SNHL) (1) is a prevalent auditory disorder caused by irreversible damage to inner ear hair cells and auditory neurons due to factors such as medications and environmental influences. Currently, there are no effective pharmacological agents in clinical practice capable of reversing its pathological state. The pathogenesis of SNHL is complex, with the core pathological processes centered around a vicious cycle involving oxidative stress, cell apoptosis, and inflammatory responses. Despite the profound understanding of its etiology in modern medicine, significant challenges remain in developing specific and multi-targeted intervention strategies.

Against this background, traditional Chinese medicine (TCM) has accumulated abundant experience in the prevention and treatment of SNHL, and its “multi-component and multi-target” action characteristics are highly compatible with the complex pathological network of SNHL. A large number of studies have demonstrated that various TCM monomers (such as resveratrol, ginsenosides, berberine, etc.) and compound prescriptions (such as Compound Jian’er Decoction and Zishen Huoxue Decoction) can exhibit hearing protection potential in experimental models by scavenging free radicals, inhibiting key apoptotic proteins, and regulating inflammatory factors. However, it should be emphasized that most of the existing studies remain at the level of pharmacodynamic observation, and direct evidence on how TCM components precisely regulate key nodes of modern molecular pathology, such as NLRP3 inflammasome activation, mitophagy, and pyroptosis, is still insufficient. The pathway network of its mechanism of action is not yet clear, which limits its translation into modern precision treatment strategies.

**Figure 1:**
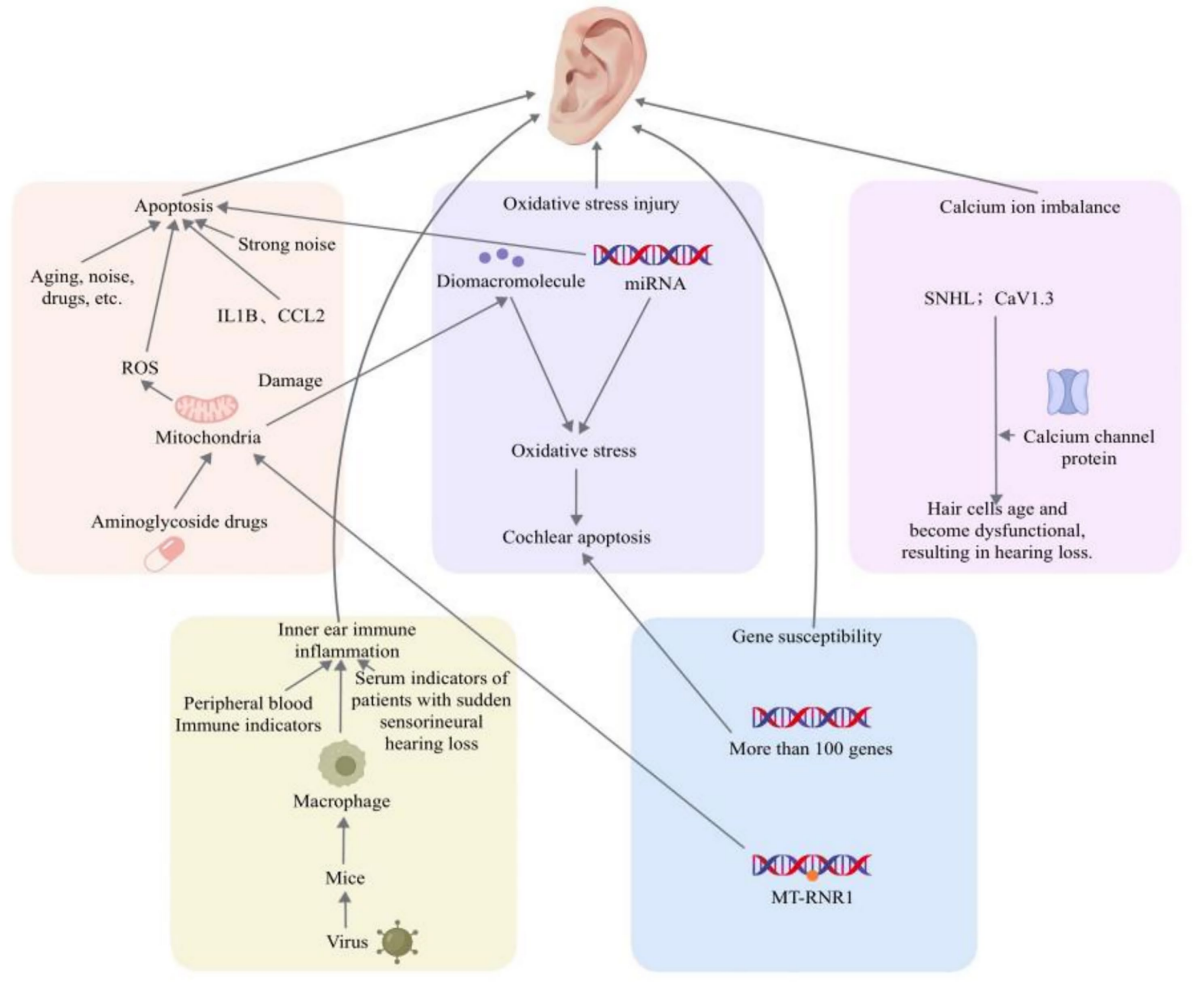
The molecular mechanism of sensorineural deafness.

Therefore, this review aims to sort out the core molecular mechanisms of SNHL (see Figure 1) and systematically review the research progress of traditional Chinese medicine (TCM) in exerting protective effects by intervening in these mechanisms. At the same time, we will critically appraise the limitations of current studies, focusing on the core problem of the weak evidence chain of molecular pathological mechanisms behind the “multi-target” advantage, in order to provide directions for future in-depth mechanism and rigorously designed research.

## 2 The molecular mechanism of sensorineural deafness

SNHL is mainly characterized by hearing loss caused by acoustic wave sensing disorders, which is closely related to the structural and functional damage of the cochlear sensory epithelium and helical neurons. At the cellular and molecular level, apoptosis, oxidative stress damage, immune inflammation, metabolic disorders, gene mutations and other factors can all lead to damage and death of cochlear cells, thereby causing deafness (Figure 1).

### 2.1 Apoptosis

Apoptosis is a process that requires energy and is initiated by specific pathways within cells. It is one of the death pathways that cells can follow when subjected to stress. Apoptosis is a key factor in SNHL, and aging of the body, noise exposure, and drug poisoning can all promote apoptosis (2–5). Immediately after exposure to intense impulse noise, the direct mechanical effect generated by the powerful liquid vortex and the secondary metabolic damage factors prompt intracellular death signals to induce changes in mitochondrial membrane permeability, ultimately leading to endogenous and exogenous apoptosis of the cells. Aminoglycoside drugs exhibit ototoxicity by destroying mitochondria, which in turn produce reactive oxygen species, leading to hair cell death and hearing loss (6). Chen et al. (7) found that in mouse models of sensorineural deafness caused by aging, noise exposure, and cisplatin ototoxicity, overexpression of IL1B and CCL2 in SNHL increased the production of reactive oxygen species, which may lead to cochlear apoptosis in SNHL.

### 2.2 Oxidative stress injury

Reactive oxygen species (ROS), as byproducts of aerobic metabolism, can cause oxidative damage to proteins, lipids, and DNA upon excessive accumulation, leading to cellular oxidative stress, which is a central mechanism underlying cochlear hair cell injury. In sensorineural hearing loss (SNHL), the body’s endogenous antioxidant defense system often fails to maintain the balance between ROS generation and clearance, resulting in irreversible damage to hair cells. Additionally, oxidative stress following miRNA-promoted apoptosis can also contribute to hearing impairment (8).

Recent studies have revealed novel mechanisms of antioxidant action (9–11). In terms of signaling pathway regulation, beyond modulating inflammatory and stress-related pathways, antioxidants can also target emerging redox-sensitive signaling pathways, such as regulating signaling molecules within the thioredoxin system. In the context of epigenetic regulation, they can modulate the activity of histone-modifying enzymes, thereby influencing gene transcription. Furthermore, antioxidants can regulate autophagy-related pathways to maintain intracellular homeostasis.

Latest advances in hearing loss protection research (12) include the development of a dual-modal antioxidant HN@TK-lipid hydrogel, which scavenges ROS through both classical and emerging pathways and modulates the NF-κB/MAPK pathway to protect cochlear hair cells. In drug-induced ototoxicity models, traditional Chinese medicines have also demonstrated hearing-protective effects through newly identified mechanisms (13).

Mitochondria serve as the primary source of ROS, and their dysfunction is closely associated with SNHL (14). Certain components of traditional Chinese medicine can regulate the balance of mitochondrial fusion and fission (15, 16). For instance, shikonin activates the Nrf2-ARE signaling pathway, reducing oxidative stress levels and thereby ameliorating sensorineural hearing impairment in mice. However, most current studies are based on *in vitro* cell models, necessitating further validation using conditional gene knockout mice.

### 2.3 Imbalance of calcium ions

Calcium ions, as the second messenger widely present in eukaryotic cells, participate in many physiological processes. Calcium ion channel proteins are important proteins for maintaining the balance of calcium ion concentration in cells. Their main functions include controlling the influx of calcium ions into cells and depolarization of cell membranes, ensuring the conduction of nerve impulses and the normal physiological functions of the inner ear. Research has found (17) that calcium ion homeostasis may be involved in the occurrence and development of SNHL, which is related to the participation of calcium ion unidirectional transporters in maintaining the structure and function of hair cells and band synapses. In cell research (18), it was found that the down-regulation of CaV1.3 promotes hair cell senescence, oxidative stress and mitochondrial dysfunction, and contributes to hearing loss.

### 2.4 Inner ear immune inflammation

The inner ear is an organ with immune response capabilities. When inflammatory reactions occur within the inner auricular blood-labyrinth barrier due to pathological factors such as noise and viruses, the number of immune cells such as neutrophils and macrophages in the inner ear immune system continuously increases to cope with inner ear damage (19). In the pathophysiological studies of SNHL, macrophages in the inner ear are a potential target for regulating the immune response in the inner ear (20). Zhang Shejiang et al.’s research (21) indicates that the elevated serum calponin level and the decreased albumin level in patients with sudden SNHL are associated with the degree of hearing loss and poor prognosis in these patients, and may serve as an auxiliary assessment indicator for the prognosis of patients with sudden SNHL. In addition, peripheral blood immune indicators can be attempted to be measured as diagnostic indicators. In an animal experiment (22), it was found that mice infected with cytomegalovirus developed hearing impairment.

The macrophages within the inner ear are not a functionally homogeneous population; they exhibit high plasticity and can polarize into distinct phenotypes in response to microenvironmental signals, playing a “double-edged sword” role in the onset and progression of SNHL. Classically activated M1 macrophages primarily release pro-inflammatory cytokines such as IL-1β, TNF-*α*, and CCL2, exacerbating tissue damage and cellular apoptosis. In contrast, alternatively activated M2 macrophages exert anti-inflammatory effects, promote tissue repair, and facilitate the clearance of apoptotic cells. In the early stages of SNHL induced by factors such as noise, ototoxic drugs, or aging, there is often a marked accumulation and activation of M1-polarized macrophages within the inner ear.

IL-1β and CCL2, as key molecules bridging innate immunity and the inflammatory cascade, play a central role in the pathological process of SNHL. Traditional Chinese medicine (TCM) has demonstrated unique advantages in interfering with the expression of these pro-inflammatory factors and chemokines.

IL-1β acts as the “primary igniter” of cochlear inflammation. Stimulated by factors such as noise or ototoxic drugs, activated macrophages release IL-1β, which subsequently activates the Caspase-1-mediated pyroptosis pathway. This directly leads to hair cell death and disrupts the integrity of the blood-labyrinthine barrier. Research indicates (23) that certain TCM formulas or monomers with heat-clearing, detoxifying, and blood-activating properties, such as berberine, can effectively block the maturation and secretion of IL-1β by inhibiting the assembly and activation of the NLRP3 inflammasome, thereby the inflammatory cascade.

CCL2, also known as monocyte chemoattractant protein-1, is a key chemokine responsible for recruiting peripheral macrophages to infiltrate the site of cochlear injury. In the early stages of damage, CCL2 is highly expressed in cochlear supporting cells and inflammatory cells, recruiting circulating monocytes to the lesion and further amplifying the local immune response. Studies have found (24) that regulating cochlear inflammation by negatively regulating the expression of MCP-1/CCL2 in cochlear fibroblasts can alleviate cochlear tissue damage.

In summary, by regulating the M1/M2 macrophage polarization balance and targeting the inhibition of the IL-1β/CCL2-mediated inflammatory cascade, TCM holds promise for breaking the vicious cycle of inner ear inflammation from an immunomodulatory perspective, thus preserving auditory function. Future, more in-depth research focusing on the inner ear microenvironment is needed to clarify its specific targets and signaling networks, providing a more precise scientific basis for the application of TCM in the treatment of SNHL.

### 2.5 Genetic susceptibility

Gene mutation is one of the causes of hearing impairment (25). According to a report by the World Health Organization (26), approximately 60% of childhood deafness is caused by genetic factors. Clinically, neonatal hearing tests can be combined with deafness gene screening on the basis of hearing screening, which can improve the diagnosis rate of neonatal hearing impairment (27). There are a large number of genes related to deafness. Currently, it is known that more than 100 genes are associated with hereditary deafness, and the connexin gene family is the most common gene causing hearing loss. Mutations that occur in GJB2, GJB6 and GJA1 (28) may all lead to generalized or incomplete hereditary deafness in newborns. The SLC26A4 (29) gene mutation is associated with large vestibular chasm syndrome. Patients with this type of deafness are particularly sensitive to head injuries or changes in pressure. The MYO7A (30) gene mutation is associated with Usher syndrome type I, a form of deafness accompanied by retinitis pigmentosa. Gene mutations in CDH23 (31) are associated with Usher syndrome type I and non-syndromic deafness. The OTOF (32) gene encodes a protein that is very important for the function of auditory hair cells, and its mutation can lead to deafness. The genes TMC1/TMC2 (33) are related to the function of mechanically gated channels, and their mutations can lead to deafness.

Mutations in mitochondrial DNA (mtDNA) are also closely related to hearing loss (34). As an important site for energy production and oxygen metabolism, mitochondria often produce a large number of oxygen free radicals. Oxygen free radicals are important mediators of inflammatory response damage, ischemic tissue damage, and intracellular metabolic damage caused by chemical drugs. MT-RNR1 is a mitochondrial gene, and its mutations are associated with aminoglycoside antibiotic sensitivity and non-syndromic deafness.

## 3 Link between the molecular mechanisms of SNHL and traditional Chinese medicine (TCM) treatment

In recent years, the use of TCM to treat hearing disorders by regulating molecular mechanisms has gradually become a research hotspot. A large number of studies have confirmed that TCM exhibits significant efficacy in hearing treatment. By reviewing relevant literature in recent years, this article summarizes TCM-based treatment for hearing loss based on the aforementioned molecular mechanisms, aiming to provide a theoretical reference for TCM intervention in deafness and the research and development of new TCM drugs.

### 3.1 TCM monomers

Resveratrol is a polyphenolic organic compound. It can affect the prognosis of SNHL through anti-inflammatory effects and may improve hair cell apoptosis caused by inflammatory factors via multi-target intervention involving TNF, CASP3, AKT1, and TP53 (35, 36). Low-dose resveratrol inhibits RIPK3-mediated necroptosis in the aging cochlea and delays the onset of age-related hearing loss, which may be closely associated with the alleviation of oxidative stress and inflammation (37). Piceatannol, a hydroxylated analog of resveratrol, can significantly mitigate HEI-OC-1 cell damage through the caspase-11-GSDMD pathway, reduce the expression of inflammation-related proteins, and inhibit pyroptosis.

Galangin is a natural flavonoid compound isolated from the roots of *Alpinia officinarum* Hance. Studies (38) have shown that galangin can act as an antioxidant and anti-apoptotic agent to prevent oxidative stress induced by aminoglycosides, thereby alleviating hearing loss. Its mechanism may involve protecting against mitochondrial dysfunction by reducing the production of mitochondrial reactive oxygen species (ROS).

Silymarin is a natural active component derived from *Silybum marianum* (L.) Gaertn. (Asteraceae) and possesses antioxidant, anti-inflammatory, and glucose metabolism-regulating properties. In a D-galactose-induced aging rat model for preventing age-related hearing loss, silymarin at a dose of 100 mg/kg/day was found to exert a protective effect against age-related hearing loss. It can be supplemented in the diet of the elderly to slow the progression of age-related hearing loss, as it has potent antioxidant and anti-aging effects (39). An animal experiment (40) demonstrated that silymarin can protect the guinea pig cochlea from hearing loss caused by temporary and permanent noise exposure.

Rosmarinic acid (RA) is a phenolic compound. Dansam-Eum (DSE) and its component RA have been proven to inhibit apoptosis of the cochlear organ of Corti and auditory cells. The underlying mechanism involves suppressing apoptosis by inhibiting ROS generation, cytochrome c release, caspase activation, AIF translocation, Bax upregulation, Bcl-2 downregulation, NF-κB activation, and IL-1β production (41). Quercetin is a flavonoid component of rosemary; it can be regulated in both intracellular and extracellular signaling pathways and exhibits anti-inflammatory, antiviral, anticancer activities, as well as the ability to prevent and treat cardiovascular and cerebrovascular diseases (42).

Puerarin is an injectable preparation extracted from the dried roots of *Pueraria montana var. lobata* (Willd.) Sanjappa & Predeep (Fabaceae). A study (43) constructed a puerarin-sudden deafness target protein–protein interaction (PPI) network using the STRING database, revealing that puerarin exerts therapeutic effects on sudden deafness through multiple targets and pathways.

Ginseng (*Panax ginseng* C.A. Meyer) belongs to the Araliaceae family. Modern pharmacological studies have shown that it has anti-diabetic, anti-tumor, anti-aging, immunity-enhancing, and cardiovascular protective effects. A study by Sun Xianchang et al. (44) found that ginsenosides can protect guinea pigs against cisplatin-induced auditory damage, and the mechanism may involve antioxidant and anti-apoptotic effects, as well as activation of the Akt-Nrf2 signaling pathway. Ginsenosides protect outer hair cells and spiral ganglia by inhibiting ROS-induced apoptosis, thereby counteracting cisplatin-induced auditory dysfunction. They also significantly increase the expression levels of phosphorylated Akt (p-Akt) and nuclear Nrf2. Therefore, the antioxidant and auditory protective effects of ginsenosides may be associated with the activation of this pathway, which in turn increases the expression of antioxidant enzymes in the cochlea. Although ginsenosides have demonstrated protective potential against cisplatin-induced auditory damage in preclinical studies, their clinical application faces significant pharmacokinetic challenges. As a class of glycosidic compounds, ginsenosides require traversal across the highly selective Blood-Labyrinth Barrier (BLB) to achieve effective therapeutic concentrations in the inner ear target tissues following systemic administration (e.g., oral or injection). Currently, research on the intracochlear distribution, metabolism, and elimination characteristics of specific ginsenoside components (such as Rb1 and Rg1) remains insufficient, and the efficiency and specific transport mechanisms for crossing the BLB are also unclear. This lack of pharmacokinetic information limits the optimization of their formulation and dosing regimen design. Future studies should focus on the cochlear pharmacokinetics of ginsenosides and explore novel delivery strategies, such as developing nanocarrier systems or combining them with permeation enhancers, to improve their intracochlear bioavailability and targeting.

Rhodiola (*Rhodiola rosea* L.) refers to the dried roots and rhizomes of plants in the Crassulaceae family. Salidroside, the main active component of Rhodiola (along with its aglycone tyrosol), exhibits anti-oxidative stress, anti-apoptotic, and anti-inflammatory effects, thereby protecting the nervous and cardiovascular systems. Experimental results by Jia Xin et al. (45) showed that salidroside can scavenge ROS, reduce the synthesis and release of inflammatory factors, inhibit inflammation, alleviate damage to auditory cortex neurons and cochlear tissues in rats with auditory deprivation, and improve their hearing. Although the auditory protective effects of salidroside have been validated in animal models, its translation from laboratory to clinical practice is hampered by its pharmacokinetic properties. As a glycoside compound with relatively high water solubility, a key challenge for salidroside is how to effectively penetrate the Blood-Labyrinth Barrier (BLB) following systemic administration. Existing research provides limited knowledge regarding its concentration-time profile in cochlear fluids, retention time within inner ear tissues, and potential metabolic pathways. These pharmacokinetic limitations make it difficult to determine its clinically effective dosage and dosing frequency. To overcome this hurdle, it is necessary to further investigate the inner ear delivery mechanisms of salidroside and evaluate local administration routes (e.g., intratympanic injection) or employ nanotechnology-based targeted delivery systems to bypass the BLB restriction, thereby achieving more efficient and safer intracochlear drug delivery.

Curculigo (*Curculigo orchioides* Gaertn.) is a plant of the Amaryllidaceae family. A study (46) showed that compared with the control group, mice treated with Curculigo extract exhibited improved prolongation of the Pa wave latency in the auditory middle latency response (AMLR) test. This suggests that Curculigo extract can alleviate cochlear, peripheral, and central auditory dysfunction induced by noise exposure in mice, and its mechanism may be attributed to the antioxidant activity of its active components.

Gastrodia elata Blume is the rhizome of a plant in the Orchidaceae family. Gastrodin, an active component of Gastrodia elata, has sedative, neuroprotective, antioxidant, and microcirculation-improving effects. A study by Yang Xiaoyu et al. (47) indicated that the total effective rate of pure tone audiometry in the gastrodin treatment group was higher than that in the control group. Periauricular injection of gastrodin exhibits significant efficacy in patients with deafness, alleviating deafness symptoms and improving quality of life.

*Ginkgo biloba* leaves are derived from *Ginkgo biloba* L. (Ginkgoaceae). They can dilate blood vessels, improve cerebral circulation, and prevent Alzheimer’s disease. A network pharmacology study by Wang Qingling et al. (48) showed that *Ginkgo biloba* extract can alleviate oxidative stress and apoptosis of spiral ganglion cells and hair cells in the cochlea through the Bcl-2/Bax pathway, thereby delaying the progression of age-related deafness and improving hearing loss. This conclusion has been preliminarily verified by animal experiments.

Propolis is a traditional natural medicine. Caffeic acid phenethyl ester (CAPE) is one of its main active components, with extensive pharmacological activities such as antiviral, antioxidant, hypoglycemic, and lipid-regulating effects. A study (49) demonstrated that CAPE can alleviate gentamicin-induced ototoxic hearing loss and protect cochlear cells from apoptosis. The mechanism involves regulating factors such as antioxidant enzymes and oxygen free radicals to protect tissues and organs against ischemia–reperfusion injury.

*Salvia miltiorrhiza* Bunge refers to the dried roots and rhizomes of plants in the Lamiaceae family. *Salvia miltiorrhiza* can increase inner ear blood flow after acoustic trauma and enhance the hypoxia tolerance of inner ear hair cells. Furthermore, a study (50) found that *Salvia miltiorrhiza* injection can promote the repair of damaged hair cells and effectively reduce the lysosomal toxicity of gentamicin to cochlear hair cells.

Chuanxiong (Ligusticum chuanxiong Hort.) is the dried rhizome of a plant in the Apiaceae family. It exhibits analgesic, anticoagulant, anti-aging, cytoprotective, and cardiac function-improving effects. Tetramethylpyrazine (TMP) is an important component of Chuanxiong; it can inhibit lipid peroxidation caused by free radical reactions by scavenging superoxide anions to a certain extent and directly eliminate cytotoxicity. A study (51) showed that TMP can upregulate the expression of autophagic proteins LC3-I and LC3-II, and downregulate the expression of autophagic protein P62 in the cochlear tissues of rats with cisplatin-induced drug-induced deafness. This promotes protective autophagy in the cochlea, thereby inhibiting cisplatin-induced hearing damage.

Stephania tetrandra S. Moore and Sinomenium acutum (Thunb.) Rehder & E.H. Wilson are both woody vines, and their rhizomes are used in TCM. Yu Y et al. (52) found that tetrandrine (an active component of Stephania tetrandra) exerts a significant protective effect on auditory function. The mechanism may involve reducing the loss of outer hair cells and cochlear synapses, as well as protecting hearing through calcium antagonism, antioxidant, and anti-inflammatory activities. A study by Zhang Qilei et al. (53) found that tetrandrine (from Sinomenium acutum) can reduce cell damage and oxidative stress responses.

Scutellaria baicalensis Georgi is a perennial herb of the Lamiaceae family. It exhibits extensive pharmacological effects such as anti-tumor and antibacterial activities. Baicalein, one of the most abundant flavonoids in Scutellaria baicalensis, can reduce cerebral vascular resistance and improve cerebral blood circulation. A study (54) found that in mice with noise-induced deafness, administration of Scutellaria baicalensis significantly reduced threshold shift, central auditory dysfunction, and cochlear functional defects, which may be associated with baicalein.

### 3.2 Traditional Chinese medicine compound

The compound ear tonic, when used in combination, jointly achieves the effects of tonifying the kidney and strengthening the spleen, soothing the liver and regulating qi, and promoting blood circulation and opening the orifices. This formula can improve microcirculation disorders, resist free radical damage and cell apoptosis, etc. The traditional Chinese medicine for tonifying qi through formula dissection can protect the auditory function impairment caused by gentamicin-induced damage to the outer hair cells of guinea pig cochlea (55). It can prevent the apoptosis pathway mediated by reactive oxygen species by activating the body and eliminating oxygen free radicals. Studies have found (56) that compound ear enhancer can intervene in the cochlea of aged mice, which is related to protecting mitochondria, preventing cyt C leakage or stabilizing cell membranes, and inhibiting the caspase pathway mediated by Fas L.

The combination of various ingredients in the Zishen Huoxue Decoction achieves the effects of tonifying the kidney and replenishing essence, strengthening the spleen and benefiting qi, and resolving phlegm and opening the orifices. Zhang Lei et al. (57) found that Zishen Huoxue Decoction has a remarkable therapeutic effect on deafness caused by kidney deficiency and blood stasis. Animal experiments have found (58) that formulas for tonifying the kidney and opening the orifices have a better therapeutic effect on hearing loss of kidney deficiency type. The mechanism is closely related to the up-regulation of signal expression and the inhibition of cell apoptosis (Table 1).

**Table 1:** Link between the molecular mechanisms of SNHL and traditional Chinese medicine (TCM) treatment.

Monomer of traditional Chinese medicine	Main function	Involving molecular mechanisms	Literature basis
Resveratrol	Anti-inflammatory, improving hair cell apoptosis caused by inflammatory factors; Inhibit the necrotic apoptosis of the aging cochlea and delay age-related hearing loss	Affects multiple targets such as TNF, CASP3, AKT1 and TP53; Improve oxidative stress and inflammation	(25–27)
Gao Liang Jiang Su	Prevent oxidative stress caused by aminoglycoside drugs and alleviate hearing loss	Reduce the production of mitochondrial ROS and protect mitochondrial function	(28)
Silymarin	Protect the cochlea from hearing loss caused by noise	Antioxidant and anti-aging	(29, 30)
Rosmarinic acid	Inhibit the apoptosis of the cochlear organ and auditory cells	It inhibits apoptosis through multiple pathways such as inhibiting the generation of reactive oxygen species and the release of cytochrome c	(31)
Quercetin	It has multiple functions such as anti-inflammation	Regulate intracellular and extracellular signaling pathways	(32)
Puerarin	Treat sudden deafness through multiple targets and multiple pathways	–	(33)
Ginsenoside	Protect guinea pigs from auditory damage caused by cisplatin	Antioxidant, anti-apoptotic, and activation of the Akt-Nrf2 signaling pathway	(34)
Salidroside	Eliminate ROS, alleviate the damage to the auditory cortex and cochlear tissue in the auditory deprivation model rats, and improve hearing	Antioxidant stress, inhibition of apoptosis, and reduction of inflammatory responses	(35)
*Curculigo orchioides* extract	Improve cochlear, peripheral and central auditory dysfunction in mice caused by noise exposure	Antioxidant	(36)
Gastrodin	Alleviate the symptoms of deaf patients and improve their quality of life	Antioxidant, etc.	(37)
Extract of *Ginkgo biloba* leaves	Relieve oxidative stress and apoptosis of cochlear spiral ganglion cells and hair cells, and delay the progression of presbycusis	The Bcl-2/Bax pathway	(38)
Phenethyl caffeic acid	Improve hearing loss caused by gentamicin ototoxicity and protect cochlear cells from apoptosis	Regulate antioxidant enzymes, oxygen free radicals, etc.	(39)
*Salvia miltiorrhiza*	Increase the blood flow in the inner ear after acoustic trauma, promote the repair of damaged hair cells, and reduce the lysosomal toxicity of gentamicin on cochlear hair cells	–	(40)
Ligustrazine	Inhibit cisplatin-induced hearing impairment	Eliminate free radicals and promote protective autophagy in the cochlea	(41)
Tetrandrine	Protect hearing function	Reduce the loss of outer hair cells and cochlear synapses, and have calcium antagonistic, antioxidant and anti-inflammatory effects	(42)
Tetrandrine	Reduce cell damage and oxidative stress responses	–	(43)
Baicalein	Reduce threshold shift, central auditory function impairment and cochlear function defects in noise-induced deafness mice	–	(44)

## 4 Summary and outlook

Sensorineural hearing loss (SNHL) is a prevalent clinical condition. Due to its diverse and complex etiological factors and pathogenic mechanisms, a complete cure remains elusive. The resulting hearing impairment imposes a significant burden on both the quality of life of affected individuals and society at large. This review systematically summarizes the key molecular mechanisms underlying SNHL, including apoptosis, oxidative stress, calcium ion imbalance, immune inflammation, and genetic susceptibility. Building upon this foundation, it highlights the therapeutic potential demonstrated by various Traditional Chinese Medicine (TCM) monomers and compounds through their intervention in these mechanisms.

However, it is crucial to acknowledge several important limitations in current research, which, to some extent, hinder the translation of findings into clinical application. Firstly, at the level of basic research, the TCM materials used in most experiments—whether monomeric components (e.g., resveratrol, ginsenosides) or compound extracts—generally lack standardized preparation processes, well-defined chemical fingerprints, and rigorous quality control. This “lack of standardization” leads to considerable variability in results between different research teams, or even across different batches from the same team, severely compromising the comparability, reproducibility, and reliability of experimental data and conclusions. Secondly, and more critically, there is a severe deficiency in clinical validation. The vast majority of current evidence regarding the use of TCM for preventing or treating SNHL originates from cell or animal model experiments. Although these preclinical studies offer valuable insights into mechanisms of action, the pathophysiological processes in animal models differ fundamentally from human disease. Furthermore, commonly employed administration routes in such studies (e.g., high-dose intraperitoneal injection) are distinct from practical clinical routes (e.g., oral administration). Presently, there is a stark lack of rigorously designed, large-scale, multicenter, randomized, double-blind, placebo-controlled clinical trials to systematically evaluate the safety, efficacy, and optimal dosing regimens of these TCM interventions in SNHL patients. This gap in high-level clinical evidence constitutes a core requirement that must be addressed before any therapy is adopted in modern evidence-based medicine, and it represents the most significant bottleneck currently impeding the clinical translation of TCM research for SNHL. Moreover, as discussed in this review, many promising TCM active ingredients (e.g., ginsenosides, salidroside) face major pharmacokinetic challenges when administered systemically, particularly in crossing the blood-labyrinth barrier (BLB). Their distribution and metabolic profiles within the inner ear remain poorly characterized. Simultaneously, the understanding of deeper mechanisms, such as the modulation of the inner ear immune microenvironment (e.g., macrophage polarization) by TCM, is still in its nascent stages.

Looking forward, to advance this field, future studies should focus on the following directions: (1) Deepening and Standardizing Basic Research: There is a need for the purification and standardization of TCM active ingredients, coupled with the integrated use of omics technologies and genetically edited animal models to elucidate their multi-target interaction networks thoroughly, especially exploring novel mechanisms like immune cell phenotypic regulation. (2) Innovating Formulations and Delivery Systems: Active development of nanocarrier-based delivery systems, polymeric materials, or local administration techniques (e.g., intratympanic injection) is warranted to overcome the BLB barrier and enhance drug targeting and bioavailability within the cochlea. (3) Conducting Evidence-Based Clinical Research: The immediate priority is to design and execute prospective clinical studies based on solid preclinical evidence, gradually progressing from case series and small-sample randomized controlled trials to large-sample multicenter studies, thereby accumulating human evidence truly applicable for clinical decision-making. (4) Promoting Multidisciplinary Integration: Achieving these goals requires deep interdisciplinary collaboration among pharmacology, pharmaceutics, otology, immunology, and clinical medicine.

In conclusion, integrating the holistic view and syndrome differentiation/treatment principles of TCM with modern molecular biology and rigorous clinical research methodologies holds promise for uncovering new therapeutic strategies for this refractory disease. By deeply revealing the scientific basis of TCM in SNHL and overcoming the limitations of current research, we can ultimately benefit a wider patient population.
